# Supplementary material for: Daily rainfall variability controls humid heatwaves in the global tropics and subtropics
Source: Nat Commun. 2025 Apr 29;16:3461. doi: 10.1038/s41467-025-58694-6 (PMC12041270; doi:10.1038/s41467-025-58694-6)
Supplement: Supplementary file 1 — Supplementary Information [file 41467_2025_58694_MOESM1_ESM.pdf]

Daily rainfall variability controls humid heatwaves in the global tropics and subtropics

Supplementary Information

Lawrence S. Jackson [1], Cathryn E. Birch [1], Guillaume Chagnaud [2], John H. Marsham [1], and Christopher M. Taylor [2, 3]

[1] School of Earth and Environment, University of Leeds, Leeds, LS2 9JT, UK. [2] UK Centre for Ecology and Hydrology, Wallingford, OX10 8BB, UK. [3] National Centre for Earth Observation, Wallingford, OX10 8BB, UK.

Corresponding author: L. S. Jackson (l.s.jackson@leeds.ac.uk)

Validation of ERA5 against station observations

We used hourly station observations from the HadISD dataset (version 3.4.0.2023f [64]) for the period 1993–2022 to validate the ERA5 data. The following steps outline the data processing workflow:

- Station Selection:

We identified 2,985 stations located between latitudes 35°S and 35°N. Of these, 1,165 stations had sufficient data to calculate daily means for all days during 1993–2022. Stations were included only if their nearest ERA5 grid cell had at least 50% land cover.

- Variables Processed:

Dry-bulb air temperature at screen height (tas); dew-point temperature at screen height (tds); and, reported sea-level pressure at screen height (psl).

- Data Aggregation

We calculated 3-Hourly Means using hourly data, requiring at least one hourly observation within each 3-hour time window. We calculated daily Means using 3-hourly means, requiring all eight 3-hourly windows to have valid data in a 24-hour period. Significant gaps in psl data necessitated the use of a reference pressure of 1,000 hPa for missing values.

- Pairing HadISD data with ERA5 Data:

Each HadISD station was paired with the nearest ERA5 grid cell. To ensure comparability, the data gaps from each HadISD station were imposed on the paired ERA5 grid cell before calculating 3-hourly and daily means.

- Calculation of Wet-Bulb Temperature:

Daily mean Twb was calculated for each station and its paired ERA5 grid cell using the same method applied in the main analysis.

Heatwave composites of net surface longwave and shortwave radiation

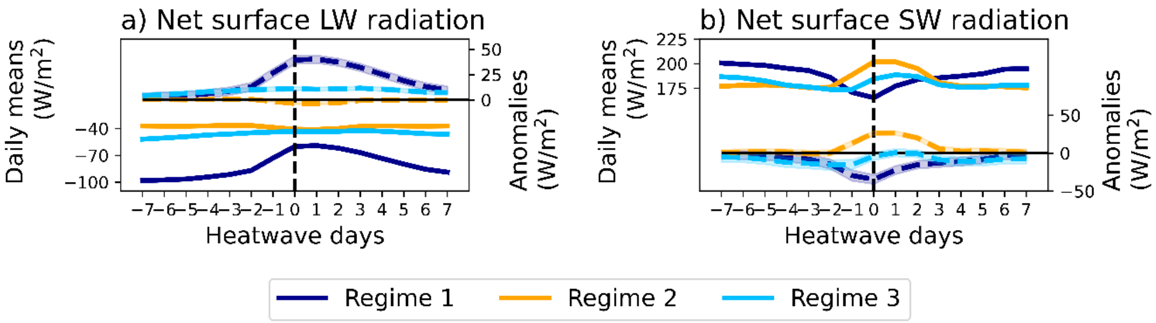

Figure S1. Heatwave composites for net surface longwave (LW) and shortwave (SW) radiation. a) Net surface LW radiation, and b) net surface SW radiation. Heatwave composite time series from 7 days before the start of each heatwave (day -7) to 7 days after the start of each heatwave (day 7). Day 0 is the heatwave start day. Solid lines and the y-axis on the left of each panel show daily mean values. Dashed lines and the y-axis on the right show anomalies from the local 1993-2022 daily mean climatology. Positive values represent fluxes from the atmosphere to the land surface. The associated surface turbulent heat and downwelling radiation fluxes are in Figure 4 of the main article.

Comparison of wet-bulb temperatures for HadISD stations and paired ERA5 grid cells

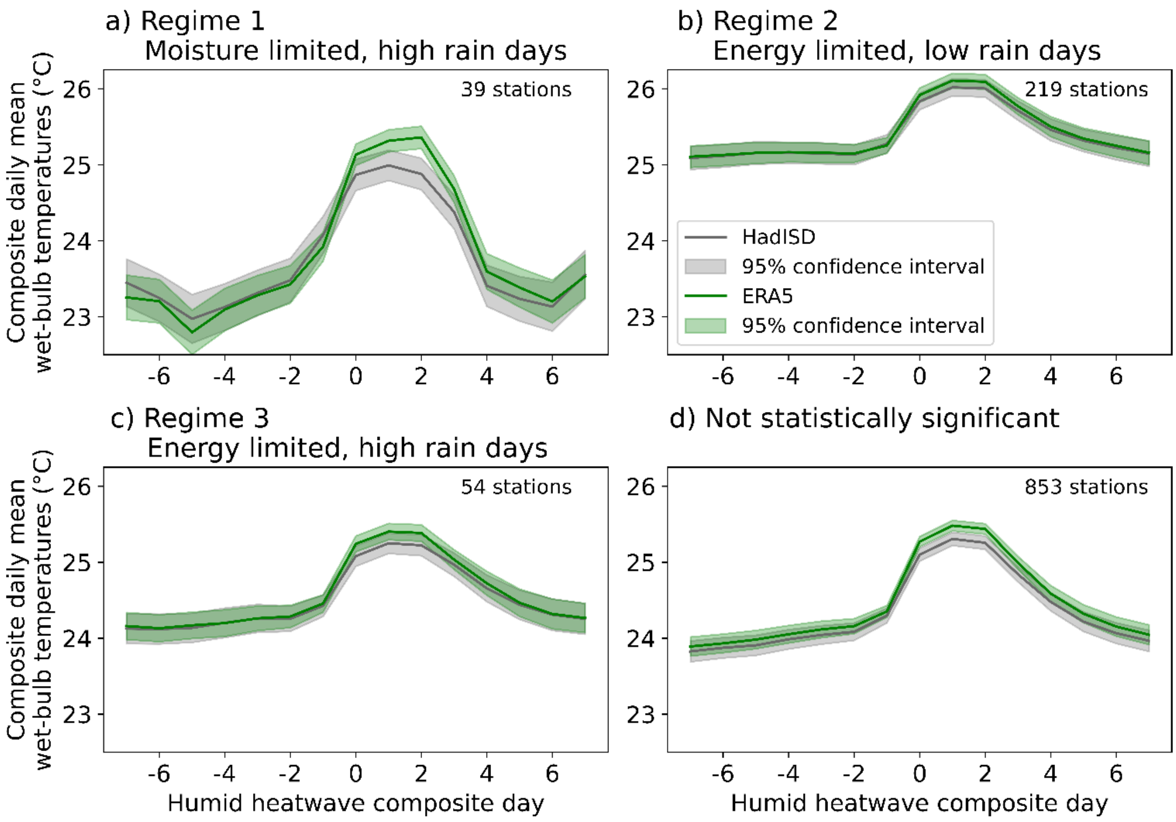

Figure S2. Heatwave composite time series for daily mean wet-bulb temperatures from selected HadISD stations and paired ERA5 grid cells (1993-2022). a) Regime 1 (moisture limited, High_Rain days), b) Regime 2 (energy limited, Low_Rain days), c) Regime 3 (energy limited, High_Rain days), and d) grid cells which are not statistically significant. The time series run from 7 days before the start of each heatwave (day -7) to 7 days after the start of each heatwave (day 7). Day 0 is the heatwave start day. The number of stations with sufficient data in each rainfall-heatwave regime is shown in the top right of each panel.

Comparison of 2m temperatures for HadISD stations and paired ERA5 grid cells

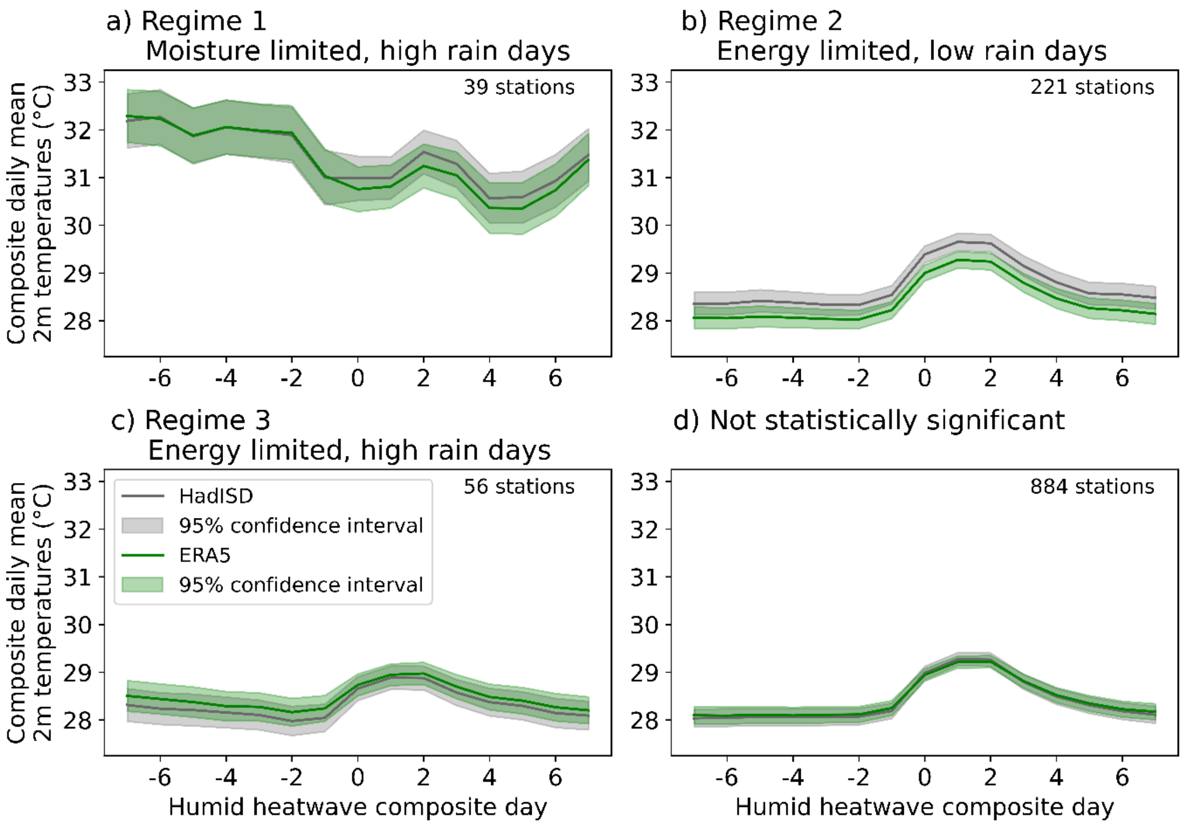

Figure S3. Heatwave composite time series for daily mean 2m temperatures from selected HadISD stations and paired ERA5 grid cells (1993-2022). a) Regime 1 (moisture limited, High_Rain days), b) Regime 2 (energy limited, Low_Rain days), c) Regime 3 (energy limited, High_Rain days), and d) grid cells which are not statistically significant. The time series run from 7 days before the start of each heatwave (day -7) to 7 days after the start of each heatwave (day 7). Day 0 is the heatwave start day. The number of stations with sufficient data in each rainfall-heatwave regime is shown in the top right of each panel.

Heatwave composite time series including grid cells that are not statistically significant

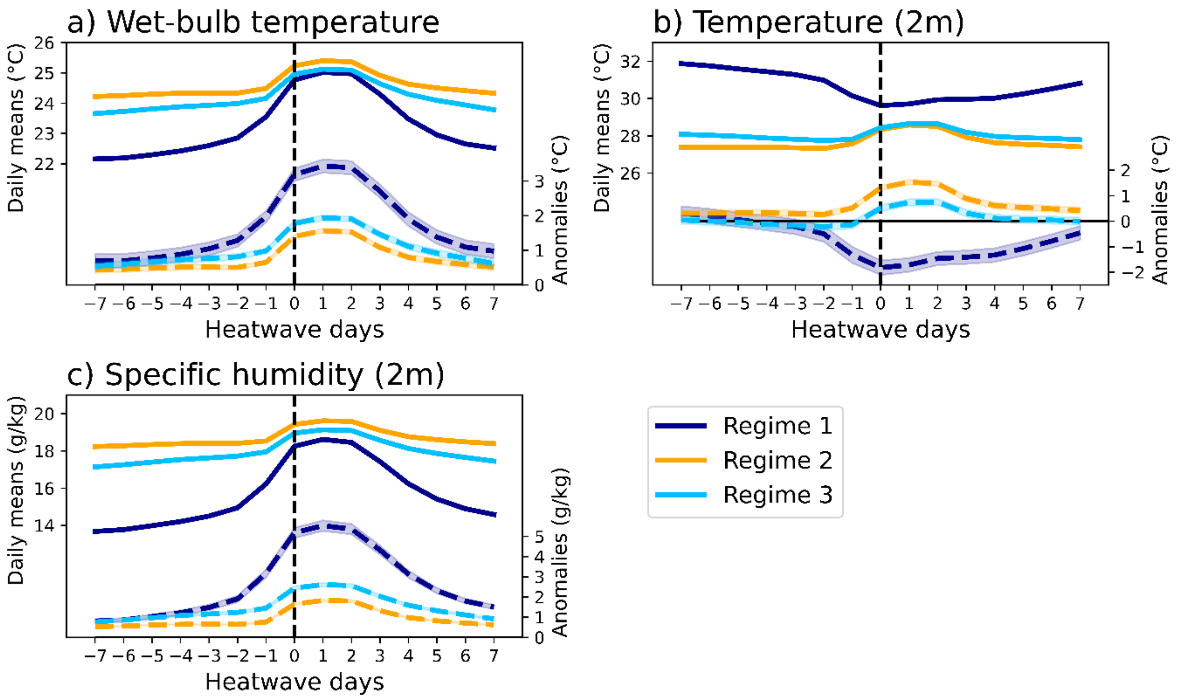

Figure S4. Heatwave composite time series including grid cells that are not statistically significant. a) Wet-bulb temperature (Twb), b) temperature (2m), and c) specific humidity (2m). The time series run from 7 days before the start of each heatwave (day -7) to 7 days after the start of each heatwave (day 7). Day 0 is the heatwave start day. Solid lines and the y-axis on the left of each panel show composite daily mean values. Dashed lines and the y-axis on the right show composite anomalies from the local 1993-2022 daily mean climatology. The shading about the dashed lines shows 95% confidence intervals for the daily anomalies. (i.e., the white regions in Figure 2d). Grid cells that are not statistically significant are assigned a regime using ELI and heatwave relative risk shown in Figure 2c. Refer to Figure 3 in the main article for the equivalent results excluding grid cells that are not significant.

Heatwave composite time series including grid cells that are not statistically significant (continued)

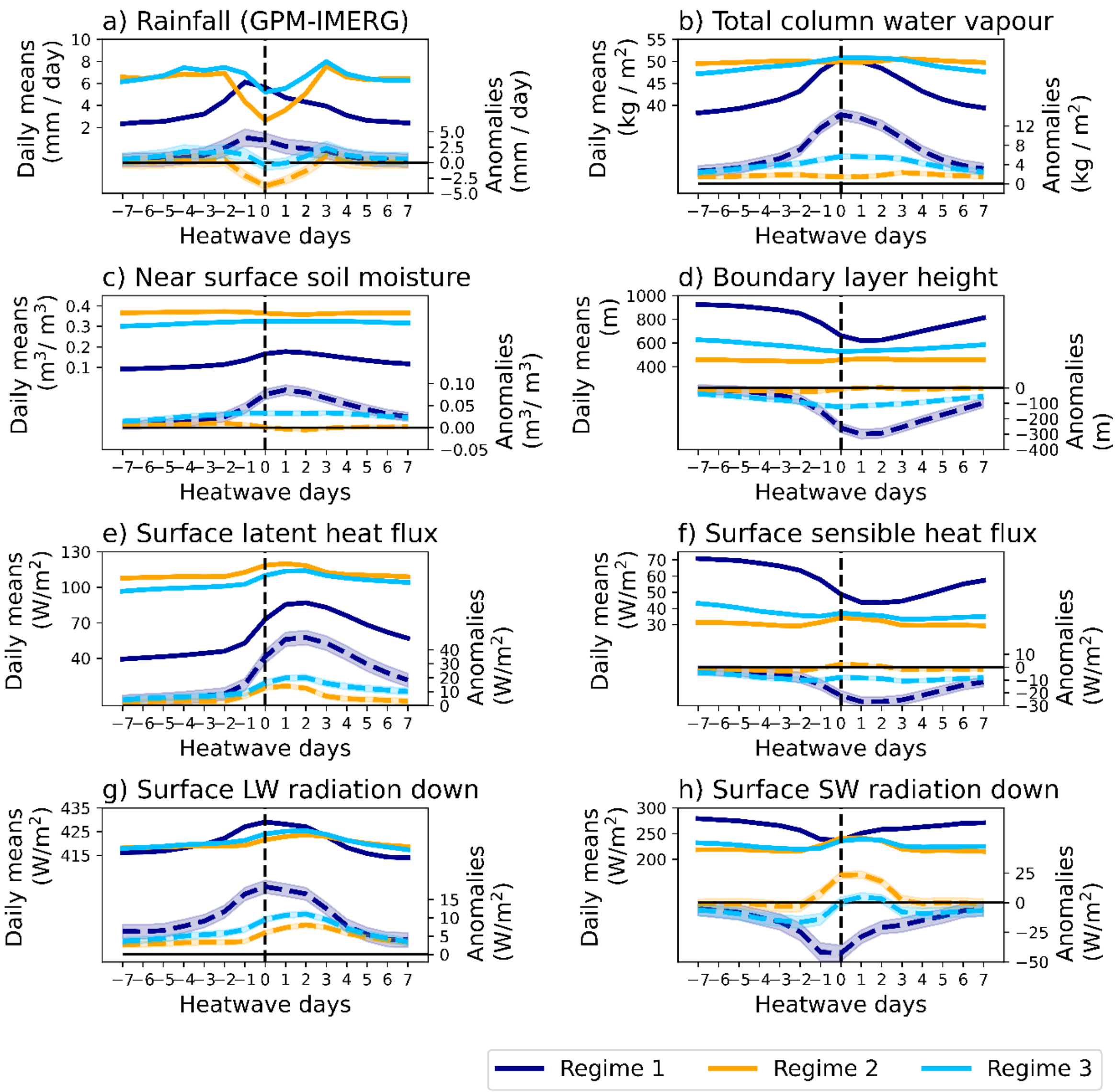

Figure S5. Heatwave composite time series including grid cells that are not statistically significant. a) Rainfall, b) total column water vapour, c) near surface soil moisture, d) boundary layer height, e) surface latent heat flux, f) surface sensible heat flux, g) surface longwave (LW) radiation down, and h) surface shortwave (SW) radiation down. Day 0 is the heatwave start day. Solid lines and the y-axis on the left of each panel show daily mean values. Dashed lines and the y-axis on the right show anomalies from the local 1993-2022 daily mean climatology. The shading about the dashed lines shows the 95% confidence intervals for the daily anomalies. Refer to Figure 4 in the main article for the equivalent results excluding grid cells that are not significant.

Heatwave and rainfall regimes using the wet-bulb temperature energy and moisture limitation index

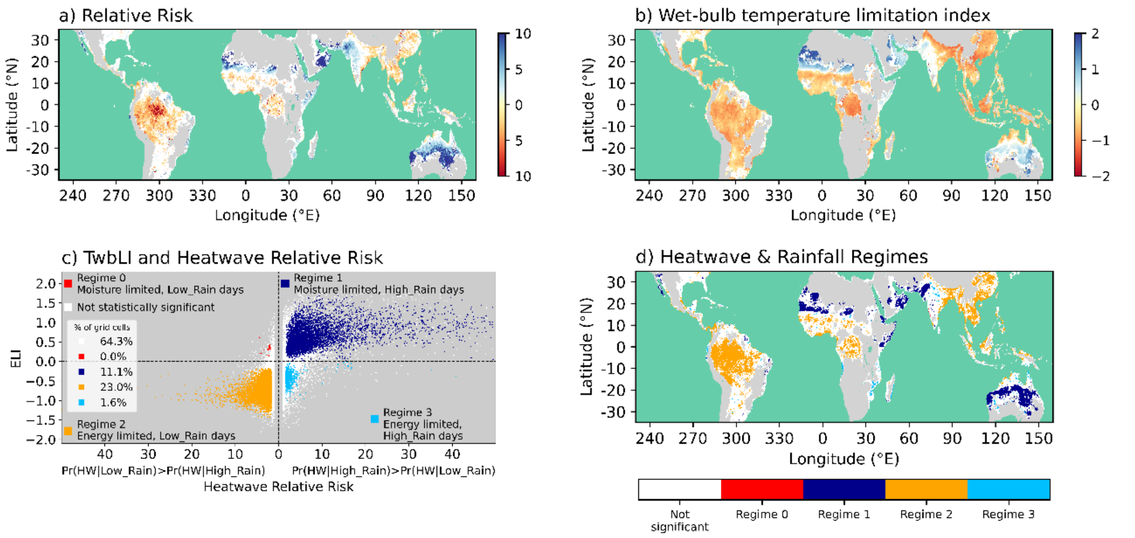

Figure S6. Heatwave and rainfall regimes using the wet-bulb temperature energy and moisture limitation index (TwbLI). a) Relative risk of heatwave occurrence on High_Rain (blue) and Low_Rain (red) days. b) TwbLI: moisture-limited regions (TwbLI > 0), energy-limited regions (TwbLI < 0). c) Scatter plot of TwbLI against relative risk and the percentage of grid cells in each regime, among all land grid cells between latitudes 35°N to 35°S where humid heatwaves occurred in 1993-2022 (inset). d) Map of the heatwave and rainfall regimes defined in panel c). In panels a, b, and d, no humid heatwaves were identified in regions shaded grey. In all panels, regions not significant at the 5% level are shaded white. Refer to Figure 2 in the main article for the equivalent results using the Ecosystem Limitation Index (ELI).

Sensitivity of relative risk to rainfall thresholds

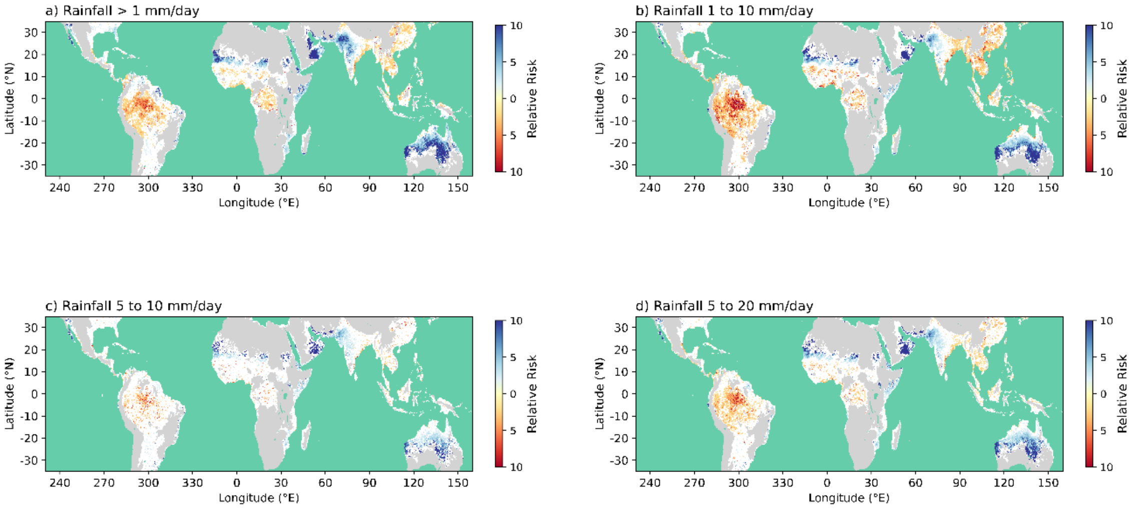

Figure S7. Sensitivity of relative risk to rainfall thresholds. a) Rainfall > 1 mm/day, b) rainfall 1–10 mm/day, c) rainfall 5-10 mm/day, d) rainfall 5-20 mm/day. Refer to Figure 2a for the equivalent results using a threshold of 5 mm/day for the definition of High_Rain and Low_Rain days.

Sensitivity of relative risk to temperature and rainfall metrics

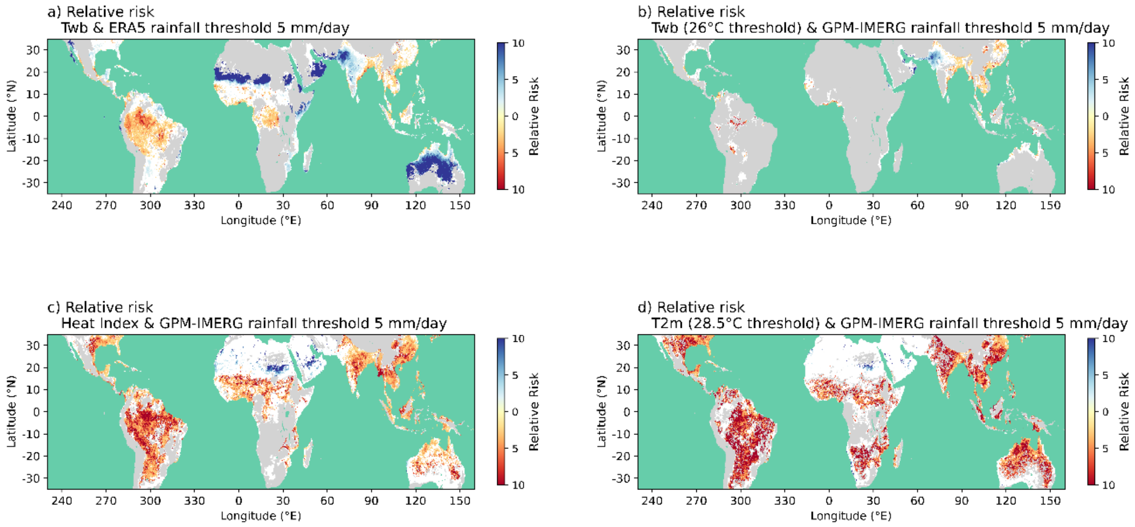

Figure S8. Sensitivity of relative risk to temperature and rainfall metrics. a) As Figure 2a except rainfall is from ERA5 (1993-2022) and the rainfall threshold is 5 mm/day. b) As Figure 2a except that heatwaves are defined using wet-bulb temperature (Twb) with a minimum threshold of 26°C instead of 24°C. GPM-IMERG with a minimum threshold of 5 mm/day is used for rainfall. c) As Figure 2a except that heatwaves are defined using the Heat Index. On heatwave days, the Heat Index exceeded the local 95th percentile and a minimum threshold of 31°C. Rainfall is from GPM-IMERG with a minimum threshold of 5 mm/day to determine High_Rain and Low_Rain days. d) As Figure 2a except that heatwaves are defined using 2m temperatures. On heatwave days, 2m temperature exceeded the local 95th percentile and a minimum threshold of 28.5°C. Rainfall is from GPM-IMERG with a minimum threshold of 5 mm/day to determine High_Rain and Low_Rain days.

Heatwave and rainfall regimes using daily maximum wet-bulb temperatures

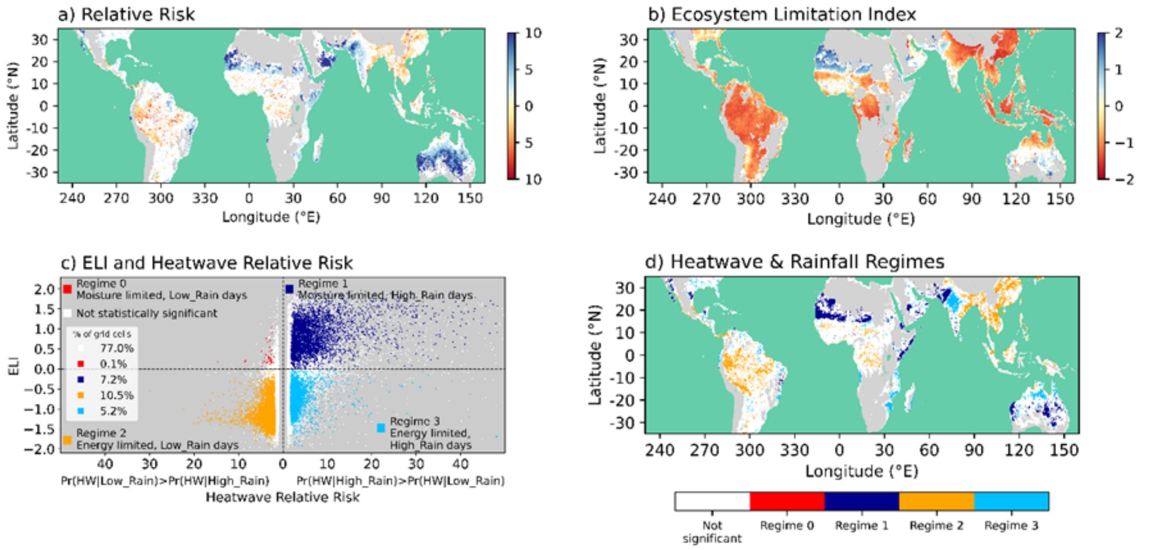

Figure S9. Heatwave and rainfall regimes using daily maximum wet-bulb temperatures (Twb). a) Relative risk of heatwave occurrence on High_Rain (blue) and Low_Rain (red) days. b) The Ecosystem Limitation Index (ELI): moisture-limited regions (ELI > 0), energy-limited regions (ELI < 0). c) Scatter plot of ELI against relative risk and the percentage of grid cells in each regime, among all land grid cells between latitudes 35°N to 35°S where humid heatwaves occurred in 1993-2022 (inset). d) Map of the heatwave and rainfall regimes defined in panel c). In panels a, b, and d, no humid heatwaves were identified in regions shaded grey. In all panels, regions not significant at the 5% level are shaded white. Refer to Figure 2 in the main manuscript for the equivalent results using daily mean Twb.
